# Hybrid Au/Si Disk-Shaped Nanoresonators on Gold Film for Amplified SERS Chemical Sensing

**DOI:** 10.3390/nano9111588

**Published:** 2019-11-08

**Authors:** Grégory Barbillon, Andrey Ivanov, Andrey K. Sarychev

**Affiliations:** 1 EPF-Ecole d’Ingénieurs, 3 bis rue Lakanal, 92330 Sceaux, France; 2 Institute for Theoretical and Applied Electrodynamics, Russian Academy of Sciences, 125412 Moscow, Russia; av.ivanov@physics.msu.ru (A.I.); sarychev_andrey@yahoo.com (A.K.S.)

**Keywords:** SERS, sensors, plasmonics, gold, silicon

## Abstract

We present here the amplification of the surface-enhanced Raman scattering (SERS) signal of nanodisks on a gold film for SERS sensing of small molecules (thiophenol) with an excellent sensitivity. The enhancement is achieved by adding a silicon underlayer for the composition of the nanodisks. We experimentally investigated the sensitivity of the suggested Au/Si disk-shaped nanoresonators for chemical sensing by SERS. We achieved values of enhancement factors of 5 × 10${}^{7}$− 6 × 10${}^{7}$ for thiophenol sensing. Moreover, we remarked that the enhancement factor (EF) values reached experimentally behave qualitatively as those evaluated with the E${}^{4}$ model.

## 1. Introduction

Surface-enhanced Raman scattering (SERS) is often employed as a fast technique of analysis owing to a high sensitivity for sensing of different types of molecules [1,2,3]. In SERS, the dominant contribution is the electromagnetic mechanism [2,4] allowing the obtaining of very high enhancement factors (EF). This EF for SERS is evaluated as the fourth power of the intensity of the local electric field [5,6]. Thus, the design of nanostructures to achieve high enhancement factors in the research domain of SERS is a very important point in order to increase the sensitivity of the biological and chemical sensing. Modern micro/nanofabrication tools such as focused ion-beam lithography [7], electron-beam lithography [8,9,10,11], X-ray, deep UV, UV, and interference lithographies [12,13,14,15,16] favor the numerous designs of SERS substrates with an accuracy control over the shape and spatial distribution of nanostructures. Furthermore, some low cost techniques of fabrication as nanoimprint lithography (NIL) [17,18] and nanosphere lithography (NSL) [19,20,21,22] may enable the realization of these SERS substrates. A large number of nanostructures such as nanodisks, nanoholes, nanodimers have been tested and provided high EFs for SERS [23,24,25]. The majority of these designs are focused on the control of the resonances of localized surface plasmons (LSPR) for optimizing the SERS enhancement [26,27]. In addition, a significant improvement of strong electric field zones around the metallic nanostructures can be observed by adding a metallic film under the plasmonic nanostructures. This enhancement is obtained thanks to the coupling between the nanostructures (antennas) via surface plasmon polaritons on the Au film [28,29] or localized surface plasmon hybridization with the image modes in a plasmonic substrate [30,31]. Thus, this supplementary enhancement can be exploited to amplify the SERS effect [21,32,33,34]. Another pathway for realizing significant EFs is to employ Si nanowires (SiNW) or nanopillars (SiNP) coupled to metallic nanoparticles or covered by a metallic layer allowing thus the obtaining of a better detection limit [35,36,37,38,39,40,41,42,43]. Moreover, fabrication techniques of large-surface may allow the realization of disordered Si nanowires. Another possibility is to realize tip-shaped Si metasurface on which metallic nanoparticles are deposited [44,45]. In addition, the Moskovits group has demonstrated that the substantial input to the SERS enhancement, for silicon/silica/metal nanogratings, is a non-local (plasmonic) effect of grating depending mainly on the grating parameters until the metal conductivity is not sufficient [46].

The main goal of this paper is to improve the SERS effect of gold nanodisks on a gold film by the simple addition of a silicon layer for the composition of the nanodisks (between the gold film and the gold layer of nanodisks). In such hybrid nanostructures, we use the second dipole resonance for enhancing the SERS signal compared to our previous works [11,41]. Moreover, these hybrid Au/Si disk-shaped nanoresonators on the gold film have been tested as chemical sensors by using solutions of thiophenol, which are small chemical molecules (thickness of a thiophenol monolayer is around 0.6 nm [47]). Besides, this additional layer of silicon has already allowed the improvement of the fluorescence signal enhanced by the surface for biosensing applications and enhanced single-molecule detection [48,49].

## 2. Experimental Details

### 2.1. Fabrication of Hybrid Au/Si Nanodisks

**Figure 1:**
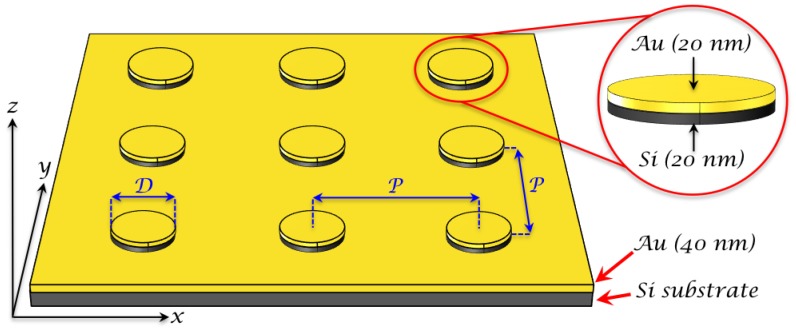
Scheme of the Au/Si disk-shaped nanoresonator array on gold film. *D* and *P* correspond to the nanodisk (ND) diameter and the period between the nanodisks, respectively. *P* is identical along *x*-axis and *y*-axis. In the red zoom are indicated the thicknesses of Si and Au layers constituting the bilayer of a hybrid nanodisk. An adhesion layer of Ti (2 nm) is used between Si substrate and gold film.

The hybrid Au/Si nanodisk (ND) fabrication is divided into several steps: (i) evaporation of a gold layer under vacuum by electron-beam (EBE) on Si substrate covered of a Ti adhesion layer for Au (2 nm), (ii) electron beam lithography, (iii) deposition of Si and Au layers, and (iv) lift-off in acetone. Firstly, a gold layer (thickness of 40 nm) was evaporated on Si substrate by EBE under normal incidence. Next, we deposited a PMMA layer (polymethylmethacrylate A2: thickness of 90 nm) by spin-coating on gold film. Then, several 300 × 300 μm${}^{2}$ arrays of nanodisks were realized by electron beam lithography (NanoBeam). Next, the sample was immersed in a development solution of 1:3 methylisobutylketone/isopropanol (MIBK/IPA) in order to reveal nanodisks. The next step consisted of an evaporation of a 20-nm silicon layer following by a second evaporation of a 20-nm gold layer both realized by EBE. Finally, a lift-off process in acetone was employed in order to obtain the hybrid Au/Si nanodisks on the gold film (see Figure 1). The evaporation rates used in this fabrication are 0.05 nm/s, 0.1 nm/s and 0.3 nm/s for Ti, Si and Au layers, respectively. In addition, geometrical parameters of hybrid nanodisks are a diameter of 130 nm (D), a period of 300 nm (P), and a total height of 40 nm (20 nm of Si + 20 nm of Au). In addition, we chose a Ti adhesion layer of 2 nm in order to have a good compromise between the adhesion properties and the electric field enhancement. Indeed, in the spectral range of our study, Ti is a material less absorptive than Cr, which is another material widely used as adhesion layer, and consequently, Ti reduces the electric field enhancement less than Cr [50].

### 2.2. Thiophenol Functionalization of the Hybrid Au/Si Nanodisks

For investigating the SERS performances of our hybrid NDs on a gold film, thiophenol molecules were used as probe molecules for their efficient grafting on metallic surfaces. The functionalization constituted of four steps: (i) realization of a thiophenol solution in ethanol (1 mM); (ii) dipping for 24 h the SERS substrate in the thiophenol solution freshly prepared (obtaining of a thiophenol monolayer on the gold parts); (iii) washing the SERS sample by using ethanol and (iv) drying it by using compressed nitrogen. For our Raman experiments in a solution which serves as reference, a highly concentrated solution of thiophenol in ethanol (1 M) was used because the Raman cross-sections of thiophenol in solution are very low.

### 2.3. Raman Spectroscopy of Hybrid Nanodisks on Gold Film

We employed a Labram spectrophotometer (Horiba Scientific) with a spectral resolution of 1 cm${}^{-1}$. For all the SERS and Raman (reference) measurements, we have set the excitation wavelength at 785 nm ($\lambda_{exc}$) and the acquisition time at 10 s. Concerning to the SERS measurements, a microscope objective (×100, N.A. = 0.9) was used in order to concentrate the laser beam on the sample. Then, SERS signal coming from the hybrid samples was detected by this same objective configured in a backscattering setup. The laser power for the excitation wavelength of 785 nm was 3 mW. Besides, for Raman measurements serving as reference, the same excitation wavelength and a macro-objective of which the focal length is 40 mm (N.A. = 0.18) were employed. All recorded spectra have been divided by the acquisition time and the laser power for comparison purposes.

### 2.4. Plasmon Resonances in Hybrid Nanodisks on Gold Film

To get inside in the plasmon resonances that are responsible for the observed SERS, we consider the plasmon resonator composed of two gold disks with a silicon layer between them (see Figure 1). Suppose, for the beginning that metal plates are optically thick, i.e., h|m|k≫1, where *h* is the metal plate thickness, $m=\sqrt{-\varepsilon_{m}}$ is the metal “refractive index”, and the wave-vector is k=2π/λ. We also suppose that the thickness *d* of the silicon layer is much smaller than the radius *a* of the resonator. Then, the distortion of the EM field near the outer boundary (r≤a) of the resonator can be neglected. The cylindrical coordinates $\left\{r,\varphi ,z\right\}$ are used below so that the *z*-axis coincides with the axis of the resonator, axes origin is in the center of the resonator. The plasmon electromagnetic field in the resonator in the dipole mode can be found from the vector potential A that has *z*-component only.

(1) $$\begin{array}{ccc} A_{z}^{\left(1\right)} & = & \exp\left[q_{2}\left(z+d/2\right)\right]J_{1}\left(qr\right)\sin\left(\varphi \right),\;-h-d/2<z<-d/2; \end{array}$$

(2) $$\begin{array}{ccc} A_{z}^{\left(2\right)} & = & \frac{\cosh\left(q_{1}z\right)}{\cosh\left(dq_{1}/2\right)}J_{1}\left(qr\right)\sin\left(\varphi \right),\;\;\;\;\;\;\;\;-d/2<z<d/2; \end{array}$$

(3) $$\begin{array}{ccc} A_{z}^{\left(3\right)} & = & \exp\left[-q_{2}\left(z-d/2\right)\right]J_{1}\left(qr\right)\sin\left(\varphi \right),\;\;\;\;z>d/2, \end{array}$$

where the silicon layer with refraction index *n* is placed in the gap −d/2<z<d/2 between two metal plates, $J_{1}\left(qr\right)$ is the Bessel function of the first order, $q_{1}=\sqrt{q^{2}-k^{2}n^{2}}$ and $q_{2}=\sqrt{q^{2}+k^{2}m^{2}}$ are the wave-vectors. The vector potentials thus defined are the solutions of the wave equations, namely, $\left(▵-{\left(mk\right)}^{2}\right)A_{z}^{\left(1,3\right)}=0$ and $\left(▵+{\left(nk\right)}^{2}\right)A_{z}^{\left(2\right)}=0$, where the symbol ▵ stands for the Laplace operator. The electric and magnetic fields in the resonator are given by the Maxwell equations $H^{\left(j\right)}=\mathrm{curl}\;A^{\left(j\right)}$, $E^{\left(j\right)}=i\mathrm{curl}\;H^{\left(j\right)}/\left[k\varepsilon^{\left(j\right)}\right]$, where *j* = 1,2,3 correspond to the upper metal plate, silicon layer, and lower metal plate correspondingly, so that $\varepsilon^{\left(1,3\right)}=\varepsilon_{m}\equiv -m^{2}$ and $\varepsilon^{\left(2\right)}=n^{2}$. Thus, at the middle plane z=0, the electric field has *z*-component only, which equals to:

(4) $$E_{z}=-i\frac{q^{2}J_{1}\left(qr\right)}{kn^{2}\cosh\left(dq_{1}/2\right)}\cos\left(\varphi \right),$$

while *z*-component of the magnetic field equals to zero everywhere. The magnetic field in the gold and silicon, obtained from vector potentials in Equations (1)–(3), has components $H_{x}$ and $H_{y}$ only in contrast to the electric field that has all three components. The magnetic field of the plasmon is perpendicular to the axis of the cylinder (*z*-axis) and plasmon can be called *HT* plasmon. Matching the fields at the metal-dielectric interfaces z=±d/2, we obtain the dispersion equation for the wave-vector *q* of the *HT* plasmon excited in the disc resonator:

(5) $$n^{2}q_{1}=m^{2}q_{2}\tanh\left(dq_{2}/2\right).$$

In a thin resonator d|n|k≪1, Equation (5) has the simple analytical solution as follows:

(6) $$q=q^{\left(s\right)}=2\;\mathrm{arctanh}\left(n^{2}/m^{2}\right)/d,$$

Therefore, the plasmon is effectively excited when metal refractive index *m* is larger in absolute value than the silicon refractive index $\left|m\right|>\left|n\right|$. The absolute value of gold permittivity is large in the optical spectral range, however, silicon permittivity ${\left|n\right|}^{2}≃15$ is also large (see [51,52,53]). Then, the condition $\left|m\right|>\left|n\right|$ is violated in the gold–silicon resonator for wavelength λ<600 nm. For smaller wavelengths, the antisymmetrical plasmon can be excited that vector potential is still given by Equations (1)–(3), where cosh(…) in Equation (2) should be replaced by sinh(…) and $A_{z}^{\left(3\right)}$ is taken with opposite sign. The dispersion equation for the antisymmetrical plasmon takes form:

(7) $$n^{2}q_{1}=m^{2}q_{2}\coth\left(dq_{2}/2\right).$$

In the thin resonator d|n|k≪1, Equation (7) has the simple analytical solution as follows:

(8) $$q=q^{\left(a\right)}=2\;\mathrm{arctanh}\left(m^{2}/n^{2}\right)/2,$$

**Figure 2:**
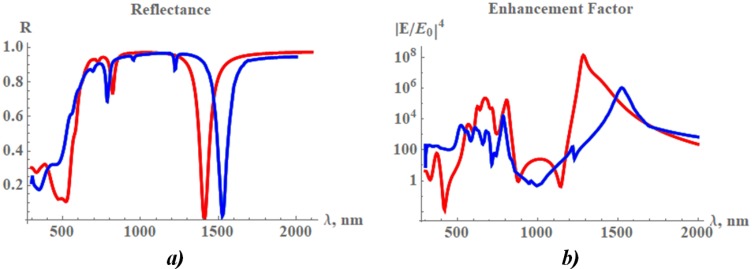
Results of analytical model (red) and COMSOL computer simulations (blue) of the system shown in Figure 1, disk diameter D=2a=130 nm, period of square lattice P=300 nm, thicknesses of the upper gold plate and silicon interlayer are 20 nm, gold film has thickness of 40 nm. (**a**) Reflectance from surface-enhanced Raman scattering (SERS) substrates and (**b**) electric field enhancement factor (EF) averaged over the lateral side of the plasmon resonator EF = $\langle |E/E_{0}{|}^{4}\rangle$, where $E_{0}$ corresponds to the amplitude of the incident EM wave.

Therefore, the antisymmetrical plasmon is effectively excited when metal refractive index *m* is smaller in absolute value than the silicon refractive index $\left|m\right|<\left|n\right|$. All plasmons discussed above could be excited simultaneously when the upper gold plate has finite thickness and radiation from the resonator cannot be neglected. Then, the energy absorption as a function of λ has set of maxima, and reflectance R(λ) has many peculiarities as displayed in Figure 2.

Suppose that the gold–silicon–gold disk resonator is illuminated from the top. The lower metal plate is still considered as optically thick. The vector potential in the resonator can be considered as a superposition of the symmetric and antisymmetric plasmons

(9) $$\begin{array}{ccc} A_{z}^{\left(1\right)} & = & \left[a_{1}\exp\left(q_{2}z\right)+a_{2}\exp\left(-q_{2}z\right)\right]J_{1}\left(qr\right)\sin\left(\varphi \right),\;-h-d/2<z<-d/2; \end{array}$$

(10) $$\begin{array}{ccc} A_{z}^{\left(2\right)} & = & \left[b_{1}\exp\left(q_{1}z\right)+b_{2}\exp\left(-q_{1}z\right)\right]J_{1}\left(qr\right)\sin\left(\varphi \right),\;\;\;-d/2<z<d/2; \end{array}$$

(11) $$\begin{array}{ccc} A_{z}^{\left(3\right)} & = & c_{2}\exp\left(-q_{2}z\right)J_{1}\left(qr\right)\sin\left(\varphi \right),\;\;\;\;z>d/2; \end{array}$$

where coefficients $a_{1},a_{2},b_{1},b_{2},c_{2}$ are obtained by matching magnetic and electric fields at the interfaces between gold and silicon at z=±d/2. We apply the boundary condition $J_{0}\left(q_{p}a\right)=0$ at the lateral boundary and found set of the harmonics *p*; $J_{0}\left(x\right)$ is the Bessel function of zero order. The incident and reflected electromagnetic waves are expanded in series of these harmonics and match the EM field in the resonator at the top of the resonator z=−h−d/2. When EM field in the resonator is known, we can calculate EM wave, which is radiated by the periodic system of the resonators shown in Figure 1. This wave is added to the wave reflected by the bare gold film on the top of the silicon substrate. Thus, obtained reflectance R(λ) is shown in Figure 2 together with results of the computer simulation.

**Figure 3:**
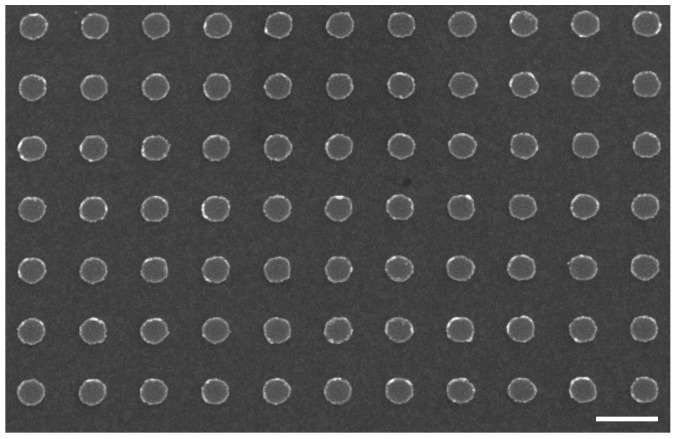
SEM picture of a hybrid Au/Si nanodisk array on gold film (scale bar = 300 nm). The nanodisk dimensions are 130 nm of diameter, 40 nm of total height, and 300 nm of periodicity.

We performed computer simulations of the periodic array of the disk resonators in the COMSOL environment. The incident light was normal to the film plane. The Maxwell equations were solved by using the finite element method (FEM). The geometrical parameters of this model are: the top nanodisk has diameter of D=2a=130 nm, thicknesses of gold and silicon disks are 20 nm. The underneath gold film had a thickness of 40 nm. The nanodisks were organized in the square lattice with a periodicity of P=300 nm (see Figure 1 and Figure 3). There was a qualitative agreement between computer simulations and the discussed simple analytical model.

**Figure 4:**
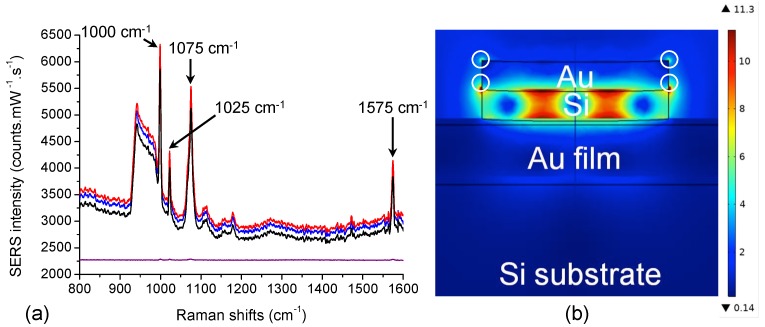
(**a**) SERS spectra of thiophenol realized on 3 distinct SERS substrates among 10 for the excitation wavelength of 785 nm. In purple is represented the SERS spectrum of thiophenol (1 mM) obtained on a 40-nm gold film at the same excitation wavelength (an offset is applied to the purple spectrum to see all the SERS spectra). (**b**) Electric field mapping $|E/E_{0}|$ of a hybrid Au/Si nanodisk on gold film for an excitation wavelength of 785 nm (cross-sectional view). White circles correspond to the strong electric field zones accessible for thiophenol molecules.

Dips in the reflectance R(λ), which are well seen in Figure 2, correspond to the various plasmon resonances. Minima at λ≃1400 nm and λ≃800 nm correspond to the first and second dipole resonances, respectively. Minima at shorter wavelengths are due to the higher symmetric as well as antisymmetric plasmon modes. Since the absolute value of the gold permittivity is on the order of the silicon permittivity, the resonances could be rather wide. The simulation results obtained for the electric field in the disk resonator are shown in Figure 4, where a dipole mode can be seen. The field spread over the entire resonator. This form of the resonance field is different from the field distribution obtained for a similar system in [54], where a 5-nm SiO${}_{2}$ layer was between the plates. We speculate the permittivity of the silica is well much lower than the absolute value of the gold permittivity and the EM field is confined in the resonator. The electric field, presented in Figure 4, is enhanced at the upper rim of the cylinder resonator. The field distribution is similar to the field calculated in [55].

Enhancement factor of the electric field averaged over lateral side of the resonator is shown in Figure 2. The enhancement $|E/E_{0}{|}^{4}$ achieves ∼10${}^{6}$ at λ≃800 nm resonance and it takes even large values ∼10${}^{8}$ at λ≃1400 nm. Reflectance R(λ) has wide minimum at λ>500 nm, however, the silicon as well as gold have large ohmic loss for λ>500 nm, and electric field is not much enhanced in this spectral band as it is seen in Figure 2b.

Since the Au film thickness is larger compared to the skin-depth (∼30 nm) and silicon plate is optically thick (opaque), the extinction (or absorbance) equals to *A* = 1−R, where the reflectance *R* is discussed above.

## 3. Results and Discussion

### 3.1. Fabrication and Functionalization of the Hybrid Disk-Shaped Nanoresonators on Gold Film

Firstly, the hybrid Au/Si disk-shaped nanoresonators on gold film have been realized by using the process of Section 2.1. A SEM picture of the obtained Au/Si nanodisks is displayed in Figure 3. The diameter, periodicity and height obtained for the hybrid NDs are 130 nm, 300 nm and 40 nm, respectively.

The next step was the functionalization of thiophenol molecules on hybrid Au/Si ND array by employing the protocol of the Section 2.2. Raman measurements were realized immediately after this functionalization step. SERS spectra of thiophenol on Au/Si nanodisks arrays on gold film obtained for the excitation wavelength of 785 nm are displayed in Figure 4a. From these spectra, Raman peaks of thiophenol molecules were observed (see refs [56,57]) of which those at 1000 cm${}^{-1}$ coincided with the association of certain modes: C–H out-of-plane bending and ring out-of-plane deformation (named: γ(CH) and r−o−d); at 1025 cm${}^{-1}$ coinciding with the association of other modes: ring in-plane deformation and C–C symmetric stretching (named: r−i−d and ν(CC)); at 1075 cm${}^{-1}$ coinciding also with the association of other modes: C–C symmetric stretching and C-S stretching (named: ν(CC) and ν(CS), respectively), and at 1575 cm${}^{-1}$ coinciding with the C–C symmetric stretching mode (named: ν(CC)). Besides, a couple of peaks located in the domain of 900–980 cm${}^{-1}$ is present and corresponding to multiphonon peaks of Si [58,59].

### 3.2. Sensitivity of the Hybrid Disk-Shaped Nanoresonators and Reproducibility of SERS Signal

For examining the detection sensitivity of hybrid Au/Si ND arrays, the EF is evaluated for the 4 previous Raman peaks by the following formula:

(12) $$\begin{array}{c} EF=\frac{I_{SERS}}{I_{Raman}}\times \frac{N_{Raman}}{N_{SERS}} \end{array}$$

where $I_{SERS}$, $I_{Raman}$ represent the SERS and Raman intensities, respectively (see Table 1). $N_{SERS}$, $N_{Raman}$ are the numbers of thiophenol molecules for SERS and reference Raman experiments, respectively. $N_{SERS}$ is determined by this formula:

(13) $$\begin{array}{c} N_{SERS}=N_{A}\times S_{illuminated}\times \sigma_{Surf} \end{array}$$

where $N_{A}$ is the Avogadro’s number (mol${}^{-1}$), $S_{illuminated}$ corresponds here to the lateral surface (gold part) of one nanodisk (ND surface: S = 8.2 × 10${}^{3}$ nm${}^{2}$) which is multiplied by the number of nanodisks (∼12) illuminated in the laser spot of which the size is about ∼1 μm${}^{2}$ for $\lambda_{exc}$ = 785 nm. $\sigma_{Surf}$ represents the surface coverage of thiophenol (here $\sigma_{Surf}$ = 0.544 nmol/cm${}^{2}$) [60,61]. Thus, thiophenol molecules of interest were grafted on lateral gold parts of hybrid nanodisks, and the number of excited molecules $N_{SERS}$ is 3.22 × 10${}^{5}$ for the excitation wavelength of 785 nm. Furthermore, no SERS signal is recorded from the smooth gold film (see Figure 4a, and as also observed in our previous works [10,11]). Moreover, we observed from the electric field mapping (see Figure 4b) that the effective SERS signals (strong electric field zones accessible for thiophenol molecules, see the white circles on Figure 4b) are localized at the interface between silicon and gold layers around the hybrid nanodisk, and also at the top of the lateral surface of the gold layer. Thus, from these observations, we speculate that the equivalent surface of interest for evaluation of EF is the lateral surface of the gold part of the hybrid nanodisk. For the Raman measurements serving as reference, the number $N_{Raman}$ is 4.24 × 10${}^{11}$ for the excitation wavelength of 785 nm. This value of $N_{Raman}$ is obtained by this expression:

(14) $$\begin{array}{c} N_{Raman}=N_{A}\times C\times V_{sca}, \end{array}$$

where *C* and $V_{sca}$ correspond to the concentration used for thiophenol molecules (1 *M*), and the scattering volume, respectively. This latter is determined by this formula: $V_{sca}$ = A × H, where A is the scattering area corresponding to the disk area with a diameter of 5.3 μm at 785 nm, and H (scattering height, see Refs [62,63]) of approximately 32 μm for $\lambda_{exc}$ = 785 nm. Thus, $V_{sca}$ is equal to 704 μm${}^{3}$∼0.704 pL.

From the results summarized in Table 1, EF values were found in the range of 5 × 10${}^{7}$–6 × 10${}^{7}$. Likewise, several groups showed good EF with similar SERS substrates composed of regular metallic nanostructures on a metallic film, such as gold nanodisks on a gold film (EF ∼ 10${}^{3}$–10${}^{4}$ in reference [8], and EF ∼ 10${}^{6}$–10${}^{7}$ in reference [11]), and 3D donut-like gold nanorings on a gold film (EF = 3.84 × 10${}^{7}$ in reference [64]). By comparing them, we remarked that our hybrid disk-shaped nanoresonators achieved higher EFs. In addition, in order to assess the substrate-to-substrate reproducibility for the SERS signal, the relative standard deviation (RSD) is evaluated for each Raman peak studied here. Each RSD value is obtained from the measurements of the SERS signal on 10 distinct substrates on which this SERS signal was recorded on 4 arrays of hybrid nanodisks (300 × 300 μm${}^{2}$) under same experimental conditions. Thus, the RSD values were obtained from 40 SERS spectra (see 3 examples in Figure 4a). Finally, a very fine substrate-to-substrate reproducibility for the SERS signal is reached for all the Raman peaks studied here (RSD ⩽5%, see Table 1).

### 3.3. Spectral Analysis

**Figure 5:**
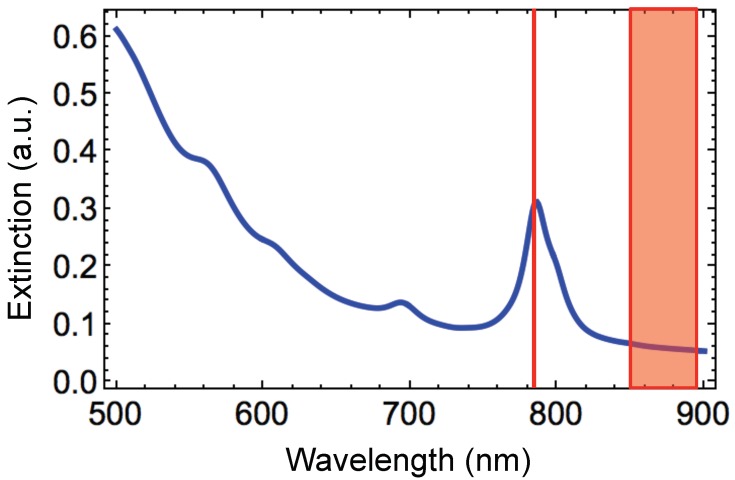
Calculated extinction spectrum of the hybrid Au/Si disk-shaped nanoresonators. The red line matches to the excitation wavelength of 785 nm. The full red rectangle represents all the Raman wavelengths ($\lambda_{Raman}$) corresponding to the associated Raman shifts (from 1000 to 1575 cm${}^{-1}$, see Table 1).

The extinction spectrum of the hybrid disk-shaped nanoresonators has been calculated by using numerical simulations (see Figure 5) in order to qualitatively compare the behavior of the experimental EF values with this of EF values obtained with the E${}^{4}$ model. The wavelengths of different resonances of hybrid NDs and the excitation and Raman wavelengths can be compared. The following expression enabled us determining the Raman scattering wavelength ($\lambda_{Raman}$):

(15) $$\begin{array}{c} \Delta \omega =10^{7}\left(\frac{1}{\lambda_{exc}}-\frac{1}{\lambda_{Raman}}\right) \end{array}$$

where Δω (cm${}^{-1}$), $\lambda_{exc}$ (nm) and $\lambda_{Raman}$ (nm) are the Raman shift, the excitation and Raman scattering wavelengths, respectively (see Table 1). In this E${}^{4}$ model, EF is presumed to be comparable to the extinction intensities (Q${}_{e}$) at $\lambda_{exc}$ and $\lambda_{Raman}$ [65] as follows:

(16) $$\begin{array}{c} EF∼Q_{e}\left(\lambda_{exc}\right)\times Q_{e}\left(\lambda_{Raman}\right). \end{array}$$

From Figure 5 and Table 1, EF${}_{1}$ corresponds to the largest value that we observed, and EFs decreased when $\lambda_{Raman}$ increased, i.e., Q${}_{e}$($\lambda_{Raman}$) decreased with $\lambda_{Raman}$. The different EF values (from EF${}_{1}$ to EF${}_{4}$) match to EFs concerning to the couples ($\lambda_{exc}$, $\lambda_{Raman1}$), ($\lambda_{exc}$, $\lambda_{Raman2}$), ($\lambda_{exc}$, $\lambda_{Raman3}$) and ($\lambda_{exc}$, $\lambda_{Raman4}$), respectively. Thus, we observed that the EFs achieved experimentally (see Table 1) behave qualitatively as those evaluated with the E${}^{4}$ model.

## 4. Conclusions

We showed the amplification of the SERS signal of nanodisks on a gold film by a simple addition of a silicon layer for the composition of the nanodisks. The sensitivity of these hybrid SERS substrates has been studied and compared to the results in literature obtained for regular gold nanostructures on a gold film. The EF values reached with the suggested SERS substrates (5 × 10${}^{7}$ < EF < 6 × 10${}^{7}$) are larger than EFs cited above. We remarked that the experimental EF values have the same behavior as those obtained with the E${}^{4}$ model by using a generic analytical approach and numerical simulations. Our hybrid Au/Si disk-shaped nanoresonators on gold film can be optimized in order to obtain even higher enhancement factors. The obtained SERS substrates offer the possibility of being incorporated on a lab-on-chip for a label-free sensor of biochemical species in the nearest future. 

## Figures and Tables

**Table 1:** For $\lambda_{exc}$ = 785 nm, and the four Raman shifts (RS) of thiophenol, $\lambda_{Raman}$ coinciding with RS, the intensities $I_{Raman}$ and $I_{SERS}$, relative standard deviations (RSDs) coinciding with $I_{SERS}$, EF obtained with Equation (1) and EF values (in arbitrary unit, see Equation (5)) calculated with the $E^{4}$ model are tabulated.

Number	RS (cm${}^{-1}$)	$\lambda_{Raman}\left(nm\right)$	$I_{Raman}$	$I_{SERS}$	RSD (%)	EF	EF (a.u.)
1	1000	852	79	3533	4.9	5.9 × 10${}^{7}$	0.0198
2	1025	854	32	1402	4.1	5.8 × 10${}^{7}$	0.0192
3	1075	857	55	2350	4.7	5.6 × 10${}^{7}$	0.0186
4	1575	896	18	724	5.0	5.3 × 10${}^{7}$	0.0170

## References

[1] Sharma B., Frontiera R.R., Henry A.-I., Ringe E., Van Duyne R.P. (2012). SERS: Materials, applications, and the future. Mater. Today.

[2] Ding S.-Y., Yi J., Li J.-F., Ren B., Wu D.-Y., Panneerselvam R., Tian Z.-Q. (2016). Nanostructure-based plasmon-enhanced Raman spectroscopy for surface analysis of materials. Nat. Rev. Mater..

[3] Barbillon G. (2017). Nanoplasmonics-Fundamentals and Applications.

[4] Wustholz K.L., Henri A.-I., McMahon J.M., Freeman R.G., Valley N., Piotti M.E., Natan M.J., Schatz G.C., Van Duyne R.P. (2010). Structure–Activity Relationships in Gold Nanoparticle Dimers and Trimers for Surface-Enhanced Raman Spectroscopy. J. Am. Chem. Soc..

[5] Itoh T., Yamamoto Y.S., Ozaki Y. (2017). Plasmon-enhanced spectroscopy of absorption and spontaneous emissions explained using cavity quantum optics. Chem. Soc. Rev..

[6] Ding S.-Y., You E.-M., Tian Z.-Q., Moskovits M. (2017). Electromagnetic theories of surface-enhanced Raman spectroscopy. Chem. Soc. Rev..

[7] Henzie J., Lee J., Lee M.H., Hasan W., Odom T.W. (2009). Nanofabrication of Plasmonic Structures. Annu. Rev. Phys. Chem..

[8] Yu Q., Guan P., Qin D., Golden G., Wallace P.M. (2008). Inverted size-dependence of surface-enhanced Raman scattering on gold nanohole and nanodisk arrays. Nano Lett..

[9] Faure A.C., Barbillon G., Ou M., Ledoux G., Tillement O., Roux S., Fabregue D., Descamps A., Bijeon J.-L., Marquette C.A. (2008). Core/shell nanoparticles for multiple biological detection with enhanced sensitivity and kinetics. Nanotechnology.

[10] Bryche J.-F., Gillibert R., Barbillon G., Sarkar M., Coutrot A.-L., Hamouda F., Aassime A., Moreau J., Lamy de la Chapelle M., Bartenlian B. (2015). Density effect of gold nanodisks on the SERS intensity for a highly sensitive detection of chemical molecules. J. Mater. Sci..

[11] Bryche J.-F., Gillibert R., Barbillon G., Gogol P., Moreau J., Lamy de la Chapelle M., Bartenlian B., Canva M. (2016). Plasmonic enhancement by a continuous gold underlayer: Application to SERS sensing. Plasmonics.

[12] Zhang P., Yang S., Wang L., Zhao J., Zhu Z., Liu B., Zhong J., Sun X. (2014). Large-scale uniform Au nanodisk arrays fabricated via X-ray interference lithography for reproducible and sensitive SERS substrate. Nanotechnology.

[13] Barbillon G., Bijeon J.-L., Lérondel G., Plain J., Royer P. (2008). Detection of chemical molecules with integrated plasmonic glass nanotips. Surf. Sci..

[14] Dhawan A., Duval A., Nakkach M., Barbillon G., Moreau J., Canva M., Vo-Dinh T. (2011). Deep UV nano-microstructuring of substrates for surface plasmon resonance imaging. Nanotechnology.

[15] Guisbert Quilis N., Lequeux M., Venugopalan P., Khan I., Knoll W., Boujday S., Lamy de la Chapelle M., Dostalek J. (2018). Tunable laser interference lithography preparation of plasmonic nanoparticle arrays tailored for SERS. Nanoscale.

[16] Hwang J.S., Yang M. (2018). Sensitive and Reproducible Gold SERS Sensor Based on Interference Lithography and Electrophoretic Deposition. Sensors.

[17] Ding T., Sigle D.O., Herrmann L.O., Wolverson D., Baumberg J.J. (2014). Nanoimprint lithography of Al Nanovoids for Deep-UV SERS. ACS Appl. Mater. Interfaces.

[18] Cottat M., Lidgi-Guigui N., Tijunelyte I., Barbillon G., Hamouda F., Gogol P., Aassime A., Lourtioz J.-M., Bartenlian B., de la Lamy Chapelle M. (2014). Soft UV nanoimprint lithography-designed highly sensitive substrates for SERS detection. Nanoscale Res. Lett..

[19] Masson J.F., Gibson K.F., Provencher-Girard A. (2010). Surface-enhanced Raman spectroscopy amplification with film over etched nanospheres. J. Phys. Chem. C.

[20] Bechelany M., Brodard P., Elias J., Brioude A., Michler J., Philippe L. (2010). Simple Synthetic Route for SERS-Active Gold Nanoparticles Substrate with Controlled Shape and Organization. Langmuir.

[21] Bryche J.-F., Tsigara A., Bélier B., Lamy de la Chapelle M., Canva M., Bartenlian B., Barbillon G. (2016). Surface enhanced Raman scattering improvement of gold triangular nanoprisms by a gold reflective underlayer for chemical sensing. Sens. Actuators B.

[22] Barbillon G., Noblet T., Busson B., Tadjeddine A., Humbert C. (2018). Localised detection of thiophenol with gold nanotriangles highly structured as honeycombs by nonlinear sum frequency generation spectroscopy. J. Mater. Sci..

[23] Brolo A.G., Arctander E., Gordon R., Leathem B., Kavanagh K.L. (2004). Nanohole-Enhanced Raman Scattering. Nano Lett..

[24] Suh J.Y., Odom T.W. (2013). Nonlinear properties of nanoscale antennas. Nano Today.

[25] Lim D.-K., Jeon K.-S., Kim H.M., Nam J.-M., Suh Y.D. (2010). Nanogop-engineerable Raman-active nanodumbbells for single-molecule detection. Nat. Mater..

[26] Félidj N., Aubard J., Lévi G., Krenn J.R., Hohenau A., Schider G., Leitner A., Aussenegg F.R. (2003). Optimized surface-enhanced Raman scattering on gold nanoparticle arrays. Appl. Phys. Lett..

[27] Guillot N., Shen H., Frémaux B., Péron O., Rinnert E., Toury T., Lamy de la Chapelle M. (2010). Surface enhanced Raman scattering optimization of gold nanocylinder arrays: Influence of the localized surface plasmon resonance and excitation wavelength. Appl. Phys. Lett..

[28] Li Z., Butun S., Aydin K. (2014). Ultranarrow Band Absorbers Based on Surface Lattice Resonances in Nanostructured Metal Surfaces. ACS Nano.

[29] Sarkar M., Besbes M., Moreau J., Bryche J.-F., Olivéro A., Barbillon G., Coutrot A.-L., Bartenlian B., Canva M. (2015). Hybrid Plasmonic Mode by Resonant Coupling of Localized Plasmons to Propagating Plasmons in a Kretschmann Configuration. ACS Photonics.

[30] Sobhani A., Manjavacas A., Cao Y., McClain M.J., Javier Garcia de Abajo F., Nordlander P., Halas N.J. (2015). Pronounced Linewidth Narrowing of an Aluminum Nanoparticle Plasmon Resonance by Interaction with an Aluminum Metallic Film. Nano Lett..

[31] Yue W., Wang Z., Whittaker J., Lopez-Royo F., Yang Y., Zayats A.V. (2017). Amplification of surface-enhanced Raman scattering due to substrate-mediated localized surface plasmons in gold nanodimers. J. Mater. Chem. C.

[32] Zhou Q., Liu Y., He Y., Zhang Z., Zhao Y. (2010). The effect of underlayer thin films on the surface-enhanced Raman scattering response of Ag nanorod substrates. Appl. Phys. Lett..

[33] Driskell J.D., Lipert R.J., Porter M.D. (2006). Labeled Gold Nanoparticles Immobilized at Smooth Metallic Substrates: Systematic Investigation of Surface Plasmon Resonance and Surface-Enhanced Raman Scattering. J. Phys. Chem. B.

[34] Mulvaney S.P., He L., Natan M.J., Keating C.D. (2003). Three-layer substrates for surface-enhanced Raman scattering: Prepartion and preliminary evaluation. J. Raman Spectrosc..

[35] Galopin E., Barbillat J., Coffinier Y., Szunerits S., Patriarche G., Boukherroub R. (2009). Silicon nanowires coated with silver nanostructures as ultrasensitive interfaces for surface-enhanced Raman spectroscopy. ACS Appl. Mater. Interfaces.

[36] Zhang M.L., Fan X., Zhou H.W., Shao M.W., Zapien J.A., Wong N.B., Lee S.T. (2010). A high-efficiency surface-enhanced Raman scattering substrate based on silicon nanowires array decorated with silver nanoparticles. J. Phys. Chem. C.

[37] He Y., Su S., Xu T.T., Zhong Y.L., Zapien J.A., Li J., Fan C.H., Lee S.T. (2011). Silicon nanowires-based highly-efficient SERS-active platform for ultrasensitive DNA detection. Nano Today.

[38] Schmidt M.S., Hübner J., Boisen A. (2012). Large area fabrication of leaning silicon nanopillars for surface enhanced Raman spectroscopy. Adv. Mater..

[39] Akin M.S., Yilmaz M., Babur E., Ozdemir B., Erdogan H., Tamer U., Demirel G. (2014). Large area uniform deposition of silver nanoparticles through bio-inspired polydopamine coating on silicon nanowire arrays for pratical SERS applications. J. Mater. Chem. B.

[40] Bryche J.-F., Bélier B., Bartenlian B., Barbillon G. (2017). Low-cost SERS substrates composed of hybrid nanoskittles for a highly sensitive sensing of chemical molecules. Sens. Actuators B.

[41] Magno G., Bélier B., Barbillon G. (2017). Gold thickness impact on the enhancement of SERS detection in low-cost Au/Si nanosensors. J. Mater. Sci..

[42] Magno G., Bélier B., Barbillon G. (2018). Al/Si nanopillars as very sensitive SERS substrates. Materials.

[43] Barbillon G. (2019). Fabrication and SERS Performances of Metal/Si and Metal/ZnO Nanosensors: A Review. Coatings.

[44] Lagarkov A., Boginkaya I., Bykov I., Budashov I., Ivanov A., Kurochkin I., Ryzhikov I., Rodionov I., Sedova M., Zverev A. (2017). Light localization and SERS in tip-shaped silicon metasurface. Opt. Express.

[45] Sarychev A.K., Ivanov A., Lagarkov A., Barbillon G. (2019). Light Concentration by Metal-Dielectric Micro-Resonators for SERS Sensing. Materials.

[46] Kanipe K.N., Chidester P.P.F., Stucky G.D., Meinhart C.D., Moskovits M. (2017). Properly Structured, Any Metal Can Produce Intense Surface Enhanced Raman Spectra. J. Phys. Chem. C.

[47] Whelan C.M., Smyth M.R., Barnes C.J. (1999). HREELS, XPS, and Electrochemical Study of Benzenethiol Adsorption on Au(111). Langmuir.

[48] Lu G., Xu J., Wen T., Zhang W., Zhao J., Hu A., Barbillon G., Gong Q. (2018). Hybrid Metal-Dielectric Nano-Aperture Antenna for Surface Enhanced Fluorescence. Materials.

[49] Zambrana-Puyalto X., Ponzellini P., Maccaferri N., Tessarolo E., Pelizzo M.G., Zhang W., Barbillon G., Lu G., Garoli D. (2019). A hybrid metal-dielectric zero mode waveguide for enhanced single molecule detection. Chem. Commun..

[50] Colas F., Barchiesi D., Kessentini S., Toury T., Lamy de la Chapelle M. (2015). Comparison of adhesion layers of gold on silicate glasses for SERS detection. J. Opt..

[51] Johnson P.B., Christy R.W. (1972). Optical Constants of the Noble Metals. Phys. Rev. B.

[52] McPeak K.M., Jayanti S.V., Kress S.J.P., Meyer S., Iotti S., Rossinelli A., Norris D.J. (2015). Plasmonic Films Can Easily Be Better: Rules and Recipes. ACS Photonics.

[53] Schinke C., Peest P.C., Schmidt J., Brendel R., Bothe K., Vogt M.R., Kröger I., Winter S., Schirmacher A., Lim S. (2015). Uncertainty analysis for the coefficient of band-to-band absorption of crystalline silicon. AIP Adv..

[54] Manjare M., Wang F., Rodrigo S.G., Harutyunyan H. (2017). Exposing optical near fields of plasmonic patch nanoantennas. Appl. Phys. Lett..

[55] Scalora M., Vincenti M.A., de Ceglia D., Grande M., Haus J.W. (2012). Raman scattering near metal nanostructures. J. Opt. Soc. Am. B.

[56] Tetsassi Feugmo C.G., Liégeois V. (2013). Analyzing the vibrational signatures of thiophenol adsorbed on small gold clusters by DFT calculations. ChemPhysChem.

[57] Li S., Wu D., Xu X., Gu R. (2007). Theoretical and experimental studies on the adsorption behavior of thiophenol on gold nanoparticles. J. Raman Spectrosc..

[58] Temple P.A., Hathaway C.E. (1973). Multiphonon Raman Spectrum of Silicon. Phys. Rev. B.

[59] Khorasaninejad M., Walia J., Saini S.S. (2012). Enhanced first-order Raman scattering from arrays of vertical silicon nanowires. Nanotechnology.

[60] Stern D.A., Wellner E., Salaita G.N., Laguren-Davidson L., Lu F., Batina N., Frank D.G., Zapien D.C., Walton N., Hubbard A.T. (1988). Adsorbed Thiophenol and Related-Compounds Studied at Pt(111) Electrodes by EELS, Auger Spectroscopy and Cyclic Voltammetry. J. Am. Chem. Soc..

[61] Caldwell J.D., Glembocki O., Bezares F.J., Bassim N.D., Rendell R.W., Feygelson M., Ukaegbu M., Kasica R., Shirey L., Hosten C. (2011). Plasmonic Nanopillar Arrays for Large-Area, High-Enhancement Surface-Enhanced Raman Scattering Sensors. ACS Nano.

[62] Alvarez-Puebla R.A. (2012). Effects of the Excitation Wavelength on the SERS Spectrum. J. Phys. Chem. Lett..

[63] Le Ru E.C., Blackie E.J., Meyer M., Etchegoin P.G. (2007). Surface enhanced Raman scattering enhancement factors: A comprehensive study. J. Phys. Chem. C.

[64] Zheng M., Zhu X., Chen Y., Xiang Q., Duan H. (2017). Three-dimensional donut-like gold nanorings with multiple hot spots for surface-enhanced Raman spectroscopy. Nanotechnology.

[65] Etchegoin P.G., Le Ru E.C., Schlücker S. (2011). Basic Electromagnetic Theory of SERS. Surface Enhanced Raman Spectroscopy: Analytical, Biophysical and Life Science Applications.

